# A Basic Architecture of an Autonomous Adaptive System With Conscious-Like Function for a Humanoid Robot

**DOI:** 10.3389/frobt.2018.00030

**Published:** 2018-04-04

**Authors:** Yasuo Kinouchi, Kenneth James Mackin

**Affiliations:** Department of Informatics, Tokyo University of Information Sciences, Chiba, Japan

**Keywords:** goal-directed behavior, habitual behavior, autonomous adaptation, image processing, binding problem, Libet’s experiment, model of consciousness, brain-oriented system

## Abstract

In developing a humanoid robot, there are two major objectives. One is developing a physical robot having body, hands, and feet resembling those of human beings and being able to similarly control them. The other is to develop a control system that works similarly to our brain, to feel, think, act, and learn like ours. In this article, an architecture of a control system with a brain-oriented logical structure for the second objective is proposed. The proposed system autonomously adapts to the environment and implements a clearly defined “consciousness” function, through which both habitual behavior and goal-directed behavior are realized. Consciousness is regarded as a function for effective adaptation at the system-level, based on matching and organizing the individual results of the underlying parallel-processing units. This consciousness is assumed to correspond to how our mind is “aware” when making our moment to moment decisions in our daily life. The binding problem and the basic causes of delay in Libet’s experiment are also explained by capturing awareness in this manner. The goal is set as an image in the system, and efficient actions toward achieving this goal are selected in the goal-directed behavior process. The system is designed as an artificial neural network and aims at achieving consistent and efficient system behavior, through the interaction of highly independent neural nodes. The proposed architecture is based on a two-level design. The first level, which we call the “basic-system,” is an artificial neural network system that realizes consciousness, habitual behavior and explains the binding problem. The second level, which we call the “extended-system,” is an artificial neural network system that realizes goal-directed behavior.

## Introduction

### Aims, Position, and Purpose of Research

In developing a humanoid robot, there are two major objectives. One is developing a physical robot having body, hands, and feet resembling those of human beings and being able to similarly control them (Jeffers and Grabowski, 2017; Tian et al., 2017). The other is to develop a control system that works similarly to our brain, to feel, think, act, and learn like ours (Dennett, 1994; Tani, 2017; Zorpette, 2017; Reggia et al., 2018). In this article, we propose an architecture as a basic logical structure of a brain-oriented control system toward realization of humanoid robot that feels, thinks, acts, and learns for the second objective. The reason for focusing on the architecture is that making the logical structure of the robot control system similar to our brain has the same important meaning as creating the physical structure of the robot resembling that of a human. The main behavioral characteristics of the humanoid robot will depend strongly on the basic logical structure of the control system.

To realize major operational characteristics of the brain in the system, we incorporate various findings from neuroscience and psychology to the proposed system. Knowledge on computer systems technology to realize highly complicated systems as well as latest artificial neural network designs is adopted at various levels to provide an integrated architecture.

Although the proposed robot’s action is primitive by focusing on clearly defining the architecture, the control system of the robot has a function similar to consciousness and autonomously adapts to the environment. As an autonomous adaptation system, the robot feels, thinks, and learns through interactions with the environment. In addition, the duality of our behavioral characteristics—habitual behavior and goal-directed behavior—which has been the subject of research in a wide field including psychology and neuroscience (Deutsch and Strack, 2006; Kahneman, 2011; Mannella et al., 2016), is also realized in the control system by adopting a two-layer logical structure.

The model of consciousness included in the architecture clearly shows that consciousness is an essential function of the parallel-processing system and proposes the method of realizing consciousness and “self” from an engineering point of view. In addition, the model is positioned as improved model of global workspace theory (GWT) (Baars, 1988; Dehaene, 2014) and explains “unity,” which is one of the basic characteristics of consciousness (Brook and Raymont, 2017).

The proposed architecture comprehensively accounts for the two major problems regarding consciousness still under debate, the time delay in Libet’s experiment (Libet, 2004), and the binding problem (Feldman, 2013). This shows that the proposed architecture is not only valid as a brain-oriented architecture but also useful as a brain model from the viewpoint of information processing. Although the function level of the robot in this article is primitive, the proposed architecture can be applied to different problems and has high scalability. By expanding on the basic architecture, it will become possible to realize a humanoid robot with both mind and body. The architecture can be useful not only for humanoid robots but also for various types of autonomous robots, in general-purpose artificial intelligence (AI) development, and for understanding the brain.

### Related Works, Methods, and Main Results

Recent developments in AI, particularly in deep learning, have shown remarkable achievements, such as mastering the game of Go (Silver et al., 2016), but current research is largely targeted toward particular fields and problems, and efforts toward brain-oriented design and human-like control systems are much smaller in comparison.

Even in the rapidly developing field of neuroscience, the whole brain’s function as a control system has yet to be clarified. The neural mechanism behind “consciousness,” a basic phenomenon of the brain, and “goal-directed behavior,” the basis of everyday behavior, are still under debate (Gremel and Costa, 2013; Hart et al., 2013; Mannella et al., 2016). Human behavior is believed to be comprised of two distinct behavior characteristics, habitual behavior and goal-directed behavior, known as the duality of human behavior (Dezfouli and Balleine, 2013). Duality in human behavior has been widely studied in many fields, for example, fast and slow thinking by Kahneman (2011) in behavioral economics, and reflective-impulsive behavior model by Deutsch and Strack (2004, 2006) in psychology, but the basic neural mechanism has not been clarified.

We have previously proposed a conceptual control system that autonomously learns and makes behavior decisions based on primitive consciousness using an artificial neural network. We had proposed a model of consciousness as a system-level function and presented an artificial neural network system that enables fast decision of optimal behavior (Kinouchi and Kato, 2013; Kinouchi and Mackin, 2015). However, our previous proposal primarily explained only habitual behavior, and goal-directed behavior could not be explained yet.

On another front, various attempts have been proposed by Franklin et al. Haikonen has proposed the Haikonen cognitive architecture (HCA) and has been operating a robot with consciousness that adapts autonomously using a neural network (Haikonen, 2003, 2007, 2012). Franklin has been running a hybrid adaptation system, Learning Intelligent Distribution Agent (Franklin and Patterson, 2006; Franklin et al., 2013, 2014). In these, the method of action decision and the model of consciousness are both developed in accordance with Baars’s proposed GWT (Baars, 1988; Baars and Franklin, 2007). In addition, Dehaene et al. proposed the global neuronal workspace that extended GWT from the viewpoint of neuroscience and tried to demonstrate it based on brain observation (Dehaene and Changeux, 2011; Dehaene, 2014). However, in these, perceptual filtering focused on only the most “salient” information is performed as an action selection based on GWT. As the salient information is not always optimal information for the system, the system’s own profits is not strictly reflected in the action selection. We assume that the basis of action decision of autonomously adaptation system is to increase the profit of the system itself as much as possible at each time. Moreover, “self” that is an essential element of consciousness should correspond to the system itself trying to make the profit as large as possible.

First, we modified, reorganized and refined our previously proposed model as a core system for efficiently realizing habitual behavior (Kinouchi, 2009; Kinouchi and Kato, 2013; Kinouchi and Mackin, 2015). Hereafter, we call this core system the “basic-system.” The basic-system autonomously adapts to the environment with functions of action decision based on profit optimization of the system at each time.

The main functions of the basic-system consist of primitive operations; (a) detecting objects from the environment and recognizing the objects, (b) action decision for the recognized objects, and (c) preparing next action including system-level learning. The importance of object handling function has been pointed out in the field of neuroscience, and then it is configured as a dedicated functional unit that enables the system to handle a bundle of signals, such as attributes of the object, collectively for processing. In action decision, an optimal action plan is calculated in a short time by using a recurrent neural network based on the Brain-State-in-a-Box (BSB), proposed by Anderson (1983) and Golden (1993). In addition, proposed circuit provides a function of powerful pattern match detection that detects matched pattern from thousands of parallel signals representing attributes of objects. This function is provided based on the findings related to pyramidal neuron (Spruston, 2008; Stuart and Spruston, 2015).

The basic-system is designed with priority on shortening response time and realized as a parallel-processing system that can quickly select desirable actions. To adapt itself to the environment, the system learns using an actor–critic reinforcement learning method, which is a kind of learning method without a teacher or a supervisor, under the control of evaluation unit incorporated in the system. Conscious phenomenon is regarded as activities for effective adaptation at the whole system-level, based on information integration and reconfiguration of individual results of the underlying paralleled functional units for preparing next action. The contents of consciousness are mainly composed of reconfigured information from attributes of the objects and evaluated value of the evaluation unit after action decision. And, these contents are transmitted to the related functional units in the whole system for speedy next action decision. These activities account for how our mind is “aware” when making our moment to moment decisions in our daily life.

Moreover, the binding problem and basic cause of the time delay in Libet’s experiment is also explained comprehensively based on the above understandings for consciousness. In explaining both the binding problem and the Libet’s delay, it is important that “the content of consciousness is reconfigured for the next action after action decision.” Furthermore, for the binding problem, it is shown that functions handling bundled signals and a powerful pattern match detection functions also play an important role.

Next, to realize goal-directed behavior, we added functions for goal management to the basic-system. Hereafter, we call this enhanced system the “extended-system.” In the extended-system, both habitual behavior and goal-directed behavior are comprehensively realized. The goal is represented and handled as a kind of object in the system, and efficient actions toward achieving the goal are successively executed.

In the extended-system, it is necessary to represent, to handle, and to recollect related reward and actions as well as the goal. To execute these functions effectively, the image handling functions are provided. In this article, we use the term “image” as “*information generated inside the system that the system can operate as an object (processing target)*” based on Haikonen (2003). Using these functions, it is possible to retrieve past experiences from long-term memory and refer to these contents for decision-making.

These operations are realized by repeated execution of the functions corresponding to the basic-system, aimed at higher reward acquisition over a long-time span. Here, consciousness is more than just “awareness” of a simple decision-making process but includes a kind of “will” or “intention” of the mind aiming at acquiring a higher level of reward, by processing sequential chains of multiple images.

## Basic Conditions and Outline of the System

### Methods and Basic Conditions of the Control System

To grasp the fundamental logical structure of the brain as easily as possible, we adopt following method. First, we assume that “the brain is a kind of information processing system that satisfies certain conditions.” Then, we clarify what functions are required, and what kind of logical configuration is necessary and efficient on the system satisfying the conditions. In this method, we do not directly imitate the structure of the brain or conscious phenomena. We expect that consciousness is designed or generated as one of the functions necessary for satisfying the system conditions; moreover, logical functions related to conscious phenomenon are totally included in the system. Based on the classification of Reggia (2013), our method is a kind of computational modeling of the “simulated consciousness” in a broad sense, but it also encompasses a part of the “instantiated consciousness.” The validity or effectiveness of the logical structure is checked based on whether or not the major characteristics of the brain can be explained using the logical structure. As the main characteristics of the brain, consciousness, and related phenomenon, binding problem, delay of Libet’s experiment, duality, etc., are used for validations.

Basic conditions of the control system are shown below.

(i) The control system autonomously adapts to the environment through learning. We consider that autonomous adaptation is the most fundamental and important system characteristic of the animal brain. To adapt itself to the environment without a teacher or a supervisor, the control system incorporates a functional unit that evaluates reward and punishment, acts under its own decision based on the evaluated value, and self-adapts based on the results of the action. As a humanoid robot control system, when the system receives a reward, the evaluation unit becomes a pleasant state, and on the other hand, when receiving a punishment, it becomes an unpleasant state. The degree of pleasant and unpleasant varies according to the degree of reward and punishment.
(ii) The system design is based on maximum performance and efficiency. The aim is not only to realize high performance but also to base the system design on maximum efficiency design. The assumption is that our brain is in a kind of optimal design state through natural selection process. By choosing maximum efficiency and optimal design from among various design possibilities, as a result, we expect that the selected design approaches that of the brain. Moreover, to realize many complexed functions with high performance, parallel processing is basically introduced.
(iii) The system is constructed by artificial neural networks. An artificial neural node is a processing element inspired by biological neural cells and is used as a basic computational element in deep learning and artificial neural networks. It is most effective from the viewpoint of high parallelism and flexible learning function. The processing speed of the element is assumed to be equivalent to an actual nerve cell.

### Operating Environment of the Robot and Basic Configuration of the System

**Figure 1:**
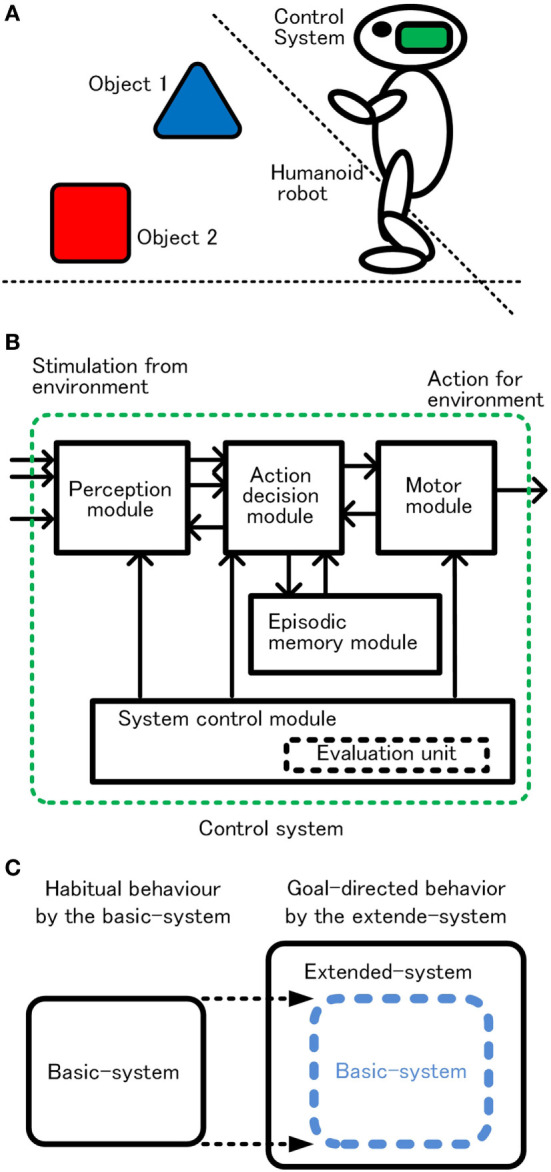
Schematic configuration of the robot in the environment **(A)** and control system **(B)**, and two step approaches for goal-directed behavior **(C)**.

Because we prioritized understanding of the basic logical structure, the control system, the robot, and the environment are limited to indispensable functions or items and set as simple as possible. The robot and its operational environment are illustrated in Figure 1A. The robot has functions that detect objects, recognize the objects, approach or avoid the objects, and earn rewards or punishments through acquisition of the objects. The robot walks randomly when there is no object in sight. When one or more objects are captured, the robot selects one preferred object and acts for it. These behaviors are controlled by the control system in the robot head. (In the following, the control system is called “system.”) Conceptual configuration of the system is shown in Figure 1B. The perception module detects and recognizes an object, and the action decision module determines an action, and the motor module executes the action. The memory module includes episodic memory. The system control module controls the operation of the whole system. Focusing on habitual behavior and goal-directed behavior, we designed the system in two stages as shown in Figure 1C. First, the basic-system realizes habitual behavior. Next, the extended-system, functional expansion of the basic-system, realizes goal-directed behavior.

## Basic Functions in the System Design

To configure an autonomous adaptive system using a neural network based on the basic conditions shown in the previous section, the following basic functions are further required.

- Handling group of signals as a bundle and handling the bundle as an object.
- Managing a signal as the signal with same meaning, even when used in various areas in the system.
- Time management by the system itself and timing adjustment of a number of parallel operating functional units consistently.

In the case of computers, these functions are usually designed and implemented based on human designer. However, in the case of an autonomous adaptive system in which the system changes the system configuration itself, these functions must be implemented as basic functions in advance. On the premise of these functions, many dedicated functional units, such as recognition and action decision function, can operate in the autonomous adaptation system.

### Handling a Group of Signals as a Bundle and Handling the Bundle as an Object

It has been reported that when animals or humans “perceive” something, inputs are selected from various stimuli to form an object, and then the object is later identified from detailed information and location information (Kahneman and Treisman, 1992; Pylyshyn, 2001; Xu and Chun, 2009). Object handling functionality has been reported to have a strong relationship with working memory features (Bays et al., 2011).

In computer systems, for effective operation, it is essential that the system can express and manage information composed of data that change over time, such as files and packets, as a bundle or a data set (Gray and Andreas Reuter, 1993; Patterson and Hennessy, 1994; Stalling, 2005). Various data or signals can be simultaneously exchanged or activated in a processor, but the data that can be processed by a program is limited to the data satisfying a specific condition, such as being on a general register or memory. For data satisfying the specific condition, a program can process the data regardless of whether that are data from an external source or internally generated data.

We have previously proposed the “object-handler” for bundling and handling information described earlier (Kinouchi and Mackin, 2015). In this article, we further clarify the functions of the object-handler for bundling signals, as well as using these bundles as an object. Only information maintained by the object-handler can be handled as an object regardless of where the signal originated from.

### Management of Signal Meaning

Information that is widely used in the system must be interpreted with the same meaning throughout the system. In this article, for information that needs to be used over a wide area or over time, a node serving as a reference of the meaning of each signal is provided, thereby managing the meaning throughout the system. Hereafter, we call this node the “reference node.” The meaning of each reference node is determined by the corresponding code conversion units, such as pattern and color recognition units.

**Figure 2:**
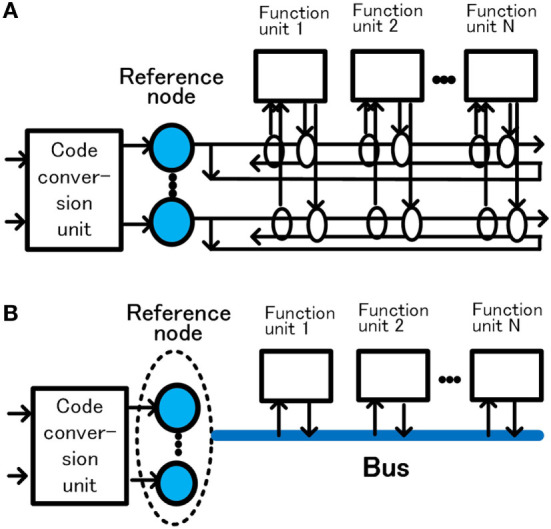
Management of signal meaning by the reference nodes and bus. Configuration of a pair of unidirectional serial connection for bidirectional transmission **(A)** and configuration represented by bus **(B)**.

When a functional unit uses signals whose meaning is already managed, the signal is supplied from the reference node. This method is based on the “*in situ*” representation proposed by van der Velde (2013). The system can be easily configured by allowing each unit to send and receive managed information bidirectionally from the reference node. In Figure 2, many functional units connected to the output of the reference node can receive signals at the same time. When one functional unit outputs a signal to the signal line, other functional units can receive the signal as a signal whose meaning is managed. In Figure 2A, a pair of unidirectional serial connection is provided for bidirectional transmission. In this way, signals whose meanings are managed by the reference node can be mutually transmitted and received between a large number of function units. Excitation of the reference node is unnecessary when merely transmitting and receiving the managed signals between the function units. Since this connection has a function similar to that of “bus” used in computers (Hwang and Briggs, 1984; Patterson and Hennessy, 1994), it is shown simplified as a bus in Figure 2B. When modifying the meaning of the reference node, a code conversion unit that determines the meaning of the reference node excites the reference node by the output of the code conversion unit.

### System Time Management

In digital computers, the time adjustment between the functional units operating in parallel is controlled using a clock running at a constant rate with high accuracy (Patterson and Hennessy, 1994). However, it is difficult to adopt this method in the system. The reason is that the processing time of the functional unit in the system is not necessarily fixed and learning for adaptation may change the processing time of the unit itself. Nonetheless, for the system to operate parallel functional units satisfying the basic condition (ii) for high performance, timing adjustment between various units is essential.

For basic timing adjustment, we used the case where the units in the system are excited simultaneously in wide-area mutual stimulation. The case indicates that the activation of each unit occurs at the same time and each unit can base the start timing from this signal. The excitement of the recurrent network for action decision described in Section “Decision Phase” provides this simultaneous excitation as a base point of timing and keeps the track of the system time by the number of iterations from this base point. However, the repetition time of this base point is long and not constant; the system subsidiarily uses together a constant period clock with short repetition time and low precision for a narrow time width. A method of dividing or slicing the clock time is also used to share the bus among various function units accessing the bus at the same time.

## Configuration and Functions of the Basic-System

**Figure 3:**
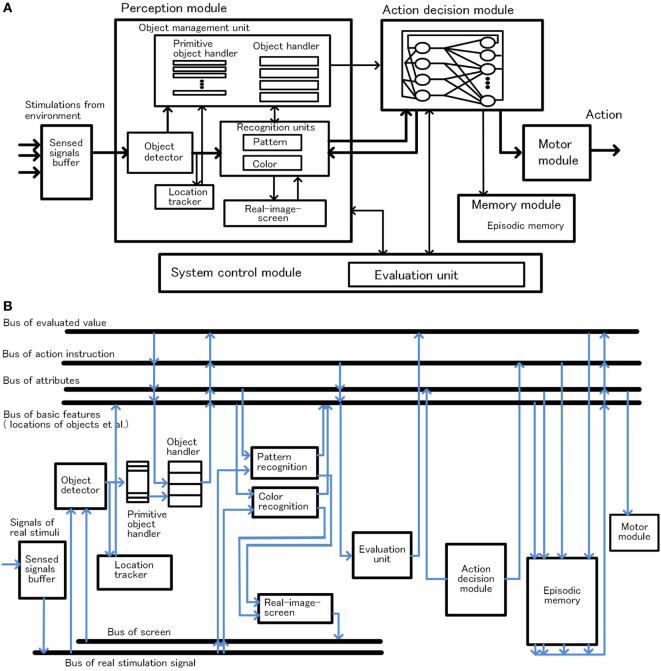
Configuration of the basic-system, module configuration in panel **(A)** and bus configuration in panel **(B)**.

**Figure 4:**
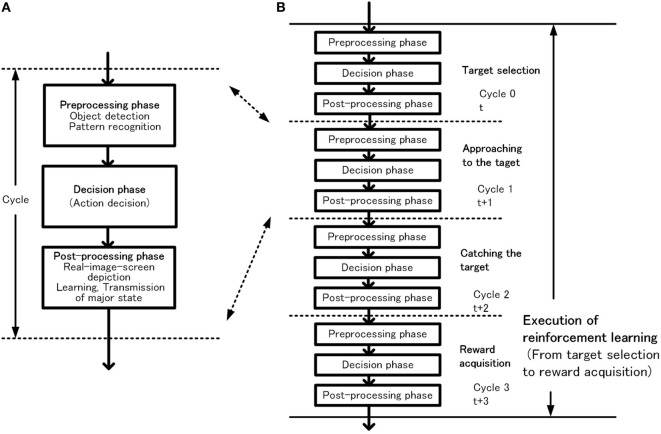
Outline of habitual behavior in the basic-system, basic processing cycle **(A)** and cycles for execution of reinforcement learning **(B)**.

In this section, the configuration of the basic-system and how habitual behavior is realized by the basic-system based on the basic conditions is described. The configuration of the basic-system is shown in Figure 3. One processing cycle of the basic-system is composed of three phases, the preprocessing phase, the decision phase, and the postprocessing phase. Through repeated iteration of the processing cycle, habitual behaviors are executed as shown in Figure 4. In the preprocessing phase, the objects are detected, and in the decision phase, action for the object is decided. The instruction for action is issued immediately after the decision phase. In the postprocessing phase, the information in the system is reorganized and prepared for the next cycle. The reason for issuing an action instruction immediately after deciding an action is that fast response to a stimulus is a major feature of habitual behavior related to the basic condition (ii). For primitive animals, the length of response time to a stimulus often becomes a matter of life or death.

The basic time of the system is counted by the number of processing cycles. Since, many networks widely excite simultaneously for action decision, the basic point for time management of the system is set at the last point in each decision phase. The execution time of one processing cycle will be simply called a cycle hereafter.

### Preprocessing Phase

Here, we describe the main process in the preprocessing phase of preparing information necessary for action decision, which consists of the following two steps:

- Detecting information to be operated by the system and managing it as a bundle of information.
- Executing pattern recognition and color recognition for bundled objects.

#### Object Detection and Management

Object detection and management are described according to Figure 3. When a group of stimuli generated in the sensed signals buffer, the object detector detects these signals as one bundle, and a primitive-object-handler in a free state captures it and sets it as a candidate of object. The primitive-object-handlers are functional units that maintain and manage temporary information of the candidate of object composed of sensed signals corresponding figure and location. From this point, the location of the candidate of object is tracked. Then, a free object-handler takes over the information of the candidate of object from the primitive-object-handler, and the object-handler starts management of the information as object. At the same time, the object-handler requests to recognize pattern or color of the object maintained in the object-handler to related functions. The object-handlers are functional units that maintain and manage the temporary information as bundles of information composed of sensed signals, location, and recognized attributes, such as pattern or color, of the object. [We assume that the primitive-object-handlers are related to function of the fragile memory, a kind of short-term memory, and the object-handlers are related to function of the working memory, based on Sligte et al. (2009, 2010); Scimeca et al. (2015); Block (2011) and Bays et al. (2011).]

Only the bundles of information managed by the object-handler can be processed for action decision by the system. This means that even if a bundle of information or signals is generated in the system itself, the bundle managed by the object-handler can be treated as an objective for action decision of the system. This method is applied to the image handling used in the extended-system. Details will be described later.

#### Object Recognition

**Figure 5:**
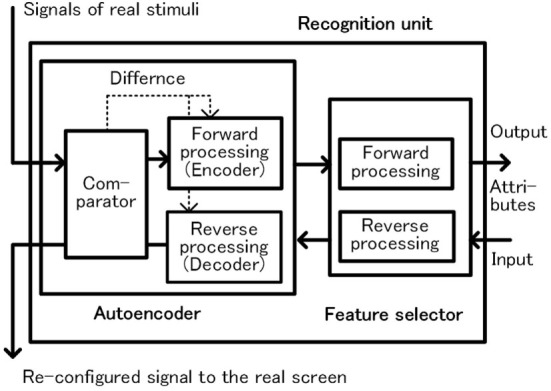
Configuration of the recognition unit.

Here, object recognition is described according to Figure 5. The object-handler instructs recognition units, such as pattern or color recognition, to recognize the object allocated to the object-handler, and maintains the attributes of the object as a result of recognition mentioned earlier. In these operations, up to four object-handlers operate concurrently in the preprocessing phase considering the capacity of the working memory (Bays et al., 2011; Block, 2011).

The recognition unit is composed of a combination of autoencoder and feature selector as shown in Figure 5. The autoencoder extracts effective features for efficient expression of the sensed signals of the object, and then the feature selector specifies the features of each attribute. An important characteristic of this unit is that it operates bidirectionally. In forward processing, the unit recognizes the sensed signals as a pattern and outputs the attribute of the pattern. In backward processing, a group of attributes are input to the recognition unit from the opposite direction, and a pattern corresponding to the group of attributes is regenerated. In this case, the feature selector reproduces the feature group from the attribute pattern. Next, the autoencoder reproduces the input pattern based on the reproduced feature group. In the preprocessing phase, the recognition unit operates only in the forward direction, and in the postprocessing phase the recognition unit operates in the reverse direction. We have currently adopted a very simple recognition function. In the field of deep learning, which is rapidly developing in recent years, combination of autoencoder and feature selector is frequently used (Ranzato et al., 2007; Bengio, 2009; Larochelle et al., 2009). We expect that this method can be applied to improve the recognition function.

### Decision Phase

#### Outline of the Decision Phase Operation

**Figure 6:**
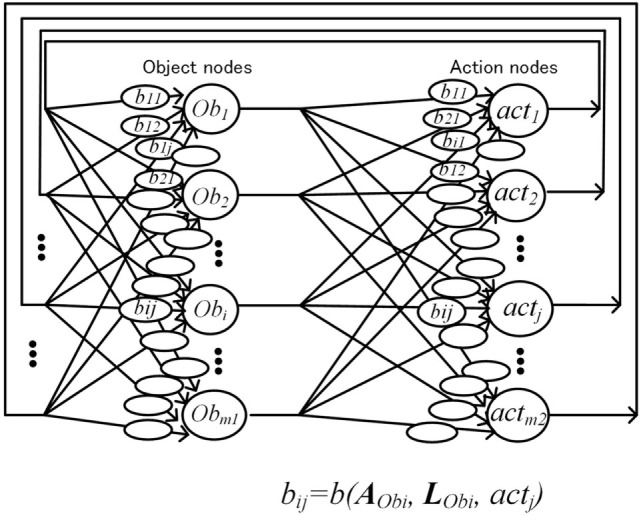
Conceptual network configuration for action decision.

In the decision phase, satisfying the basic condition (ii), the system quickly selects the most desirable pair for the system at that time from a large number of objects and action pairs, and issues the result immediately as an action instruction using the recurrent neural network in Figure 6. The configuration of the recurrent neural network is equivalent to the BSB, proposed by Anderson (1983) and generalized by Golden (1986, 1993). Golden has revealed that the BSB is a gradient descent algorithm in the direction to reduce the cost represented by the cost function (corresponding to the energy function). BSB has been studied mainly as a method for categorization.

In this article, the cost function expressed by the quadratic expression of connection weights between nodes, corresponds to the desirability of the system (system desirability *D*). As shown in the following section, by changing the connection weights according to action evaluation, such as pleasant/unpleasant, the cost function can be modified and trained by the experience of the system. By performing the steepest descent algorithm under this cost function, optimization operation for the desirability of the system is possible using a recurrent neural network.

#### Detail Processing in Decision Phase

As shown previously, each object-handler maintains attributes and location information of the assigned object. Attribute of object *i* (Ob*_i_*) is expressed by a vector ${\text{A}}_{\text{O}{\text{b}}_{i}}=\left(a_{1},a_{2},\ldots ,a_{k1}\right)$. When Ob*_i_* has corresponding micro-feature *j*, then *a_j_* = 1, and when Ob*_i_* has no corresponding micro-feature *j*, then *a_j_* = 0. Similarly, location of Ob*_i_* is expressed by a vector ${\text{L}}_{\text{O}{\text{b}}_{i}}=\left(l_{1},l_{2},\ldots ,l_{k1}\right)$. When Ob*_i_* is found at distance *l_j_*, then *l_j_* = 1, and when Ob*_i_* is not found at distance *l_j_*, then *l_j_* = 0. (For simplification, only one distance *l_j_* is set to 1 and others are set to 0.)

The operation selecting the desirable object–action pair is speedily executed by iterations based on the BSB as shown in Figure 6. The cost function is defined by system desirability *D* as expressed in Eq. 1. Variables $x_{\text{O}{\text{b}}_{i}}\left(n,t\right)$ and $y_{{\text{act}}_{j}}\left(n,t\right)$ represent the degree of how necessary or desirable object Ob*_i_* and action act*_j_* is for the system in the *n*th iteration at time *t*, and is implemented as the activation level of neural nodes, which correspond to Ob*_i_* or act*_j_*. Coefficient $b_{\mathrm{ij}}^{t}\left({\text{A}}_{\text{O}{\text{b}}_{i}},{\text{L}}_{\text{O}{\text{b}}_{i}},{\text{act}}_{j}\right)$ indicates desirability of object–action pair of object Ob*_i_* and action act*_j_*, and is implemented as the connection weights between object node *i* and action node *j*

(1) $$D\left(n,t\right)=\sum_{\text{O}{\text{b}}_{i},{\text{act}}_{j}}\;b_{\mathrm{ij}}^{t}\;\left({\text{A}}_{\text{O}{\text{b}}_{i}},{\text{L}}_{\text{O}{\text{b}}_{i}},{\text{act}}_{j}\right)x_{\text{O}{\text{b}}_{i}}\;\left(n,t\right)y_{{\text{act}}_{j}}\;\left(n,t\right)\;.$$

Activation levels of object or action nodes are increased or decreased from initial states according to *D* in a limited number of iterations. After the iteration, detecting the object and action node with maximum activation means selecting the semi-optimum object–action pair for *D* at time *t*. In the optimization process, constraints such as $\sum_{\text{O}{\text{b}}_{i}}\;{x_{\text{O}{\text{b}}_{i}}}^{2}\leq 1$ and $\sum_{{\text{act}}_{j}}\;{y_{{\text{act}}_{j}}}^{2}\leq 1$ are applied, but for simplicity, these constraints are abbreviated in this article. Operations mentioned earlier are executed along the following equations. The characteristics of neural nodes are defined by Eqs 2 and 3 with a piecewise-linear activation function

(2) $$x_{\text{O}{\text{b}}_{i}}\;\left(n+1,t\right)=f\;\left(\varphi_{i}\;\left(n,t\right)\right),$$

$$\begin{array}{lll} f\left(\varphi_{i}\;\left(n,t\right)\right)\left\{\begin{array}{c} =1\;\text{if}\;\varphi_{i}\;\left(n,t\right)>1\\ =\varphi_{i}\;\left(n,t\right)\;\\ =0\;\text{if}\;\varphi_{i}\;\left(n,t\right)<1 \end{array}\right. & \; & \end{array}$$

where

(3) $$\varphi_{i}\;\left(n,t\right)=x_{\text{O}{\text{b}}_{i}}\;\left(n,t\right)+\sum_{{\text{act}}_{j}}\;b_{\mathrm{ij}}^{t}\;\left({\text{A}}_{\text{O}{\text{b}}_{i}},{\text{L}}_{\text{O}{\text{b}}_{i}},{\text{act}}_{j}\right)y_{{\text{act}}_{j}}\;\left(n,t\right)\;.$$

Equations 4–6 are lead from Eqs 1 to 3

$$\begin{array}{lll} \frac{\Delta D\;\left(n,t\right)}{\Delta x_{\text{O}{\text{b}}_{i}}\;\left(n,t\right)}≅\sum_{{\text{act}}_{j}}\;b_{\mathrm{ij}}^{t}\;\left({\text{A}}_{\text{O}{\text{b}}_{i}},{\text{L}}_{\text{O}{\text{b}}_{i}},{\text{act}}_{j}\right)y_{{\text{act}}_{j}}\;\left(n,t\right) & \; & \end{array}$$

(4) $$x_{\text{O}{\text{b}}_{i}}\;\left(n+1,t\right)\;-\;x_{\text{O}{\text{b}}_{i}}\;\left(n,t\right)\;=\;\sum_{{\text{act}}_{j}}\;b_{\mathrm{ij}}^{t}\;\left({\text{A}}_{\text{O}{\text{b}}_{i}},{\text{L}}_{\text{O}{\text{b}}_{i}},{\text{act}}_{j}\right)\;y_{{\text{act}}_{j}}\;\left(n,t\right)\;,$$

(5) $$x_{\text{O}{\text{b}}_{i}}\;\left(n+1,t\right)-x_{\text{O}{\text{b}}_{i}}\;\left(n,t\right)≅\frac{\Delta D\;\left(n,t\right)}{\Delta x_{\text{O}{\text{b}}_{i}}\;\left(n,t\right)}\;,$$

(6) $$y_{{\text{act}}_{j}}\;\left(n+1,t\right)-y_{{\text{act}}_{j}}\;\left(n,t\right)≅\frac{\Delta D\;\left(n,t\right)}{\Delta y_{{\text{act}}_{j}}\;\left(n,t\right)}.$$

Based on above Eqs 4–6, the desirable object–action pair is selected using the gradient method in BSB.

**Figure 7:**
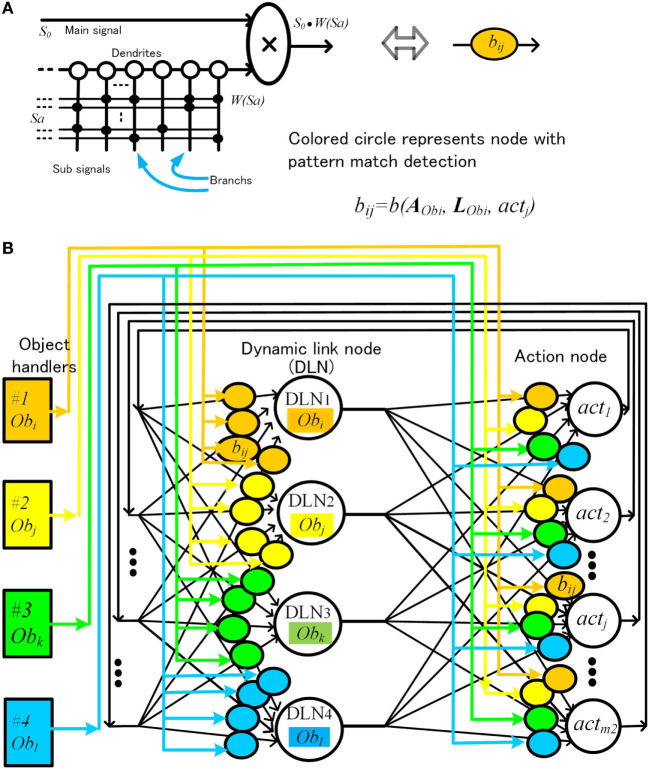
Artificial neural node with pattern match detection **(A)** and schematic configuration of action decision network using dynamic link nodes **(B)**.

The following two extensions are adopted for implementing the network to the basic-system:

- The coefficient $b_{\mathrm{ij}}^{t}\left({\text{A}}_{\text{O}{\text{b}}_{i}},{\text{L}}_{\text{O}{\text{b}}_{i}},{\text{act}}_{j}\right)$ is effective only when an object of a certain attribute is in a certain place. This means that a single neural node must be able to detect patterns of attribute and location signals on its own. Previous artificial neural models require a large network of neurons for such pattern detection. To cope with this problem, we proposed a pattern match detection method inspired by the pyramidal neurons in the cerebral cortex, in which the dendritic structure support various matching detection. One pyramidal neuron has thousands of branches in the dendrite, and each branch processes thousands of paralleled input signals (Spruston, 2008; Kasai et al., 2010; Coward, 2013).
  Schematic diagram of the artificial neural node is shown in Figure 7A. Information is composed of main signal *s*_0_ (0 ≤ *s*_0_ ≤ 1) and sub-signal ${\text{S}}_{a}=\left(s_{a1},s_{a2},\ldots ,s_{k3}\right)$. For simplicity, *s_ai_* = 0 or 1. Each branch memorizes a sub-signal pattern ***S****_a_*, where $W_{S_{a}}$ is a weight corresponding to this pattern ***S****_a_*. This pyramidal neural node outputs $s_{0}\cdot W_{S_{a}}$, only when input pattern ***S****_a_* is matched with the pattern in the branches.
- In the method shown in Figure 6, there is another disadvantage. As an object with a specific pattern of attributes and location is assigned to a fixed physical object node, the same object that has changed location is assigned as a different object. The object changed location should be treated as a same object. To achieve this, we proposed a method called dynamic link node (DLN). Schematic configuration is shown Figure 7B. In this method, we limit the number of object nodes to 4, and we make four pairs of an object-handler and an object node. Each object node represents and functions as an object maintained by the paired object-handler. Each object-handler supplies attributes and location information to the paired object nodes. This means that same physical object node can operate as different object node dynamically by changing information maintained paired object-handler.

Equations 5 and 6 are transformed as below, corresponding to Figure 7B

$$r_{k}\;\left(\text{O}{\text{b}}_{i},n+1,t\right)=r_{k}\;\left(\text{O}{\text{b}}_{i},n,t\right)+\sum_{{\text{act}}_{j}}\;b_{\mathrm{ij}}^{t}\;\left({\text{A}}_{\text{O}{\text{b}}_{i}},{\text{L}}_{\text{O}{\text{b}}_{i}},{\text{act}}_{j}\right)\;y_{{\text{act}}_{j}}\;\left(n,t\right),$$

$$y_{{\text{act}}_{j}}\;\left(n+1,t\right)=y_{{\text{act}}_{j}}\;\left(n,t\right)+\;\;\sum_{k\;\left(\text{O}{\text{b}}_{i}\right)}\;b_{\mathrm{ij}}^{t}\;\left({\text{A}}_{\text{O}{\text{b}}_{i}},{\text{L}}_{\text{O}{\text{b}}_{i}},{\text{act}}_{j}\right)\;r_{k}\;\left(\text{O}{\text{b}}_{i},n,t\right).$$

where *r_k_*(Ob*_i_, n, t*) indicates activation of DLN *k* which work as node of Ob*_i_*. ${\text{A}}_{\text{O}{\text{b}}_{i}}$ and ${\text{L}}_{\text{O}{\text{b}}_{i}}$ are supplied by the object-handler according to the object processed at that time. Although wired connection is fixed, the circuit in Figure 7B is able to process various objects dynamically.

**Figure 8:**
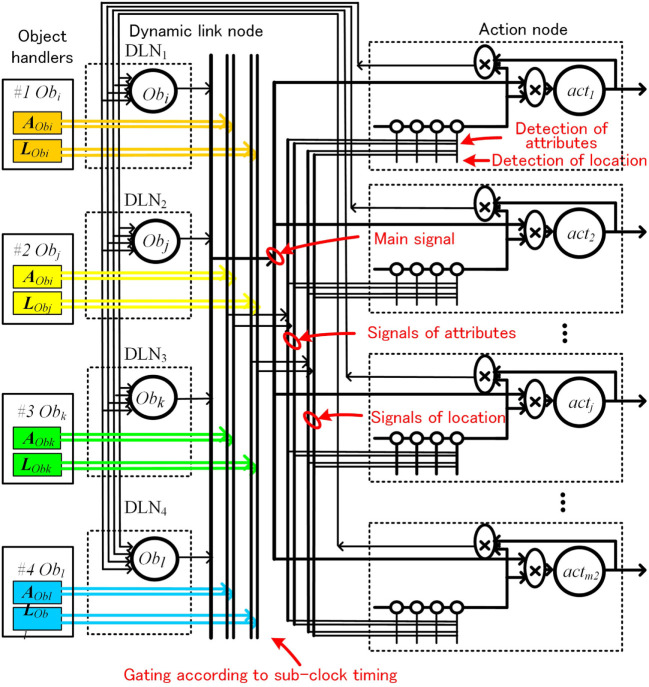
Action decision network using dynamic link node with time division control.

However, implementing the circuits according Figure 7B is not easy. As the circuits have to wire four set of attributes and location signals to nodes, the circuit becomes very complicated. To avoid this problem, we introduced a time division method, controlled by a sub-clock, which sends four sets of attributes and position information using one set of wire. The configuration is depicted in Figure 8.

### Postprocessing Phase

In the postprocessing phase, the system first reconfigures major information scattered in the system and performs necessary learning for adaptation of itself. Then, to respond quickly to new stimulus in the next cycle in line to the basic condition (ii), transmitting and processing of the major information are executed.

#### Reconfiguration of Information and Learning

The operation of the autonomous adaptation system can be described largely as two operations: (a) operations for external environment as an action of the system and (b) learning operations for the system itself to change its configuration for adaptation. For (a), the system issued an action instruction at the end of the decision phase so as to perform the action instruction at the fastest and highest priority in line to the basic condition (ii). However, for (b), it is necessary to evaluate the result of the action based on the current system at the relevant time and to instruct the related units in the system to make changes based on the evaluation. It should be noted here that a large number of function units operating in parallel in the system may cause incompatible or inconsistent states among the units.

To deal with these problems, in the postprocessing phase, first, the main states in the system are reconfigured and coordinated. The system updates the information of each object-handler to the latest one, and integrates the same object-handler as the same object when the positions overlap even if they are different object-handler. Through these processes, each object-handler has the latest information of the allocated object. Then information expressing the object’s figure with shape and color are reconfigured on the real-image-screen using the object’s attributes and location. These attributes and location maintaining by the object-handler were recognized results in the preprocessing phase and were effective for action decision. We call reconfigured information corresponding to a real object existing in environment at that time as a “real-image.” The “real-image-screen” is a kind of short-term memory, which maintains the real-images resembling real figures of objects. The reconfiguration of the real-image is performed by reverse processing of the recognition unit using the attribute maintained by the object-handler.

Almost at the same time, processing for two kinds of learning is performed. One is a learning of the recognition unit performed locally, and the other is a learning in relation to action decisions performed as a whole system. The former learning of the recognition unit is performed as the same process when the recognition units execute the reconfiguration of a real image. During reconfiguration, the autoencoder in the recognition unit in Figure 5 compares decoder’s outputs with external stimuli of the object using the comparator and executes self-learning to reduce the difference. Although the real-image-screen is drawn with the output signals of autoencoder, as each signal is checked with each real external stimulus, a highly accurate figure with shape and color can be drawn. Since the recognition units keep learning and correction in each cycle, even if the figure of the object changes slowly over time, it can be recognized as the same object. We presume that the contents of the real-image-screen correspond to what we are aware of when we are looking at things outside in daily life (Meyer and Damasio, 2009).

On the other hand, the latter, learning of action decision is executed as reinforcement learning executed in cooperation with the episodic memory. In the postprocessing phase, the system only writes information for learning into the episodic memory. This information is read later in the sleeping mode and used for learning of action decision module. In the sleeping mode, the robot is powered on, but it does not respond to external stimuli. Details are shown in the next section.

#### Transmission of System-Level-Shared-Information and Writing of Learning Information to the Episode Memory

In the latter part of the postprocessing phase, the system makes the state in the system consistent and compatible by widely transmitting and processing the major information for the efficient and speedy next cycle operation. At the same time, information for the system to learn in sleeping mode is written to episodic memory. We focus on the followings as the major information in the system and call this information “system-level-shared-information.”

- The real-image, reconfigured information of the object on the real-image-screen.
- The information of the evaluated value by the evaluation unit (pleasant/unpleasant).

The system widely transmits this information into the system and processes as follows:

(i) The information of the evaluated value and the object on the real-image-screen is sent into the system *via* the bus.
(ii) The recognition unit that receives the object information from the real-image-screen executes forward recognition processing for the object information. The recognized results, composed of attribute of the object, are transmitted into the system *via* the bus. (For example, if the red circle is on the real-image-screen as an object, “red” and “circle” attribute nodes are output by recognition unit and these attributes are transmitted *via* the bus.)
(iii) The content of object-handler is concurrently updated based on the information from the recognition unit.

In these operations, the reference node corresponding to the meaning of each signal is also excited. As a result, based on the transmission and processing using the system-level-shared-information, the state of each unit connected to the buses and the reference nodes, which are provided parallel in the system, are set in a consistent and compatible state. On these consistent and compatible states, next cycle operation of the parallel units can be executed efficiently and speedy related to basic condition (ii).

The episode memory is connected to the main buses, such as buses related to attributes, action, etc., as shown in Figure 3B, and forms a record by collecting information of these buses. The record mainly consists of information reorganized on the bus based on the system-level-shared-information and action instructions. Writing to the episodic memory is executed at the end of the postprocessing phase.

## Learning Process for Action Decision in Basic-System

This section describes the learning process in the basic-system of the robot.

### Execution of Reinforcement Learning

**Figure 9:**
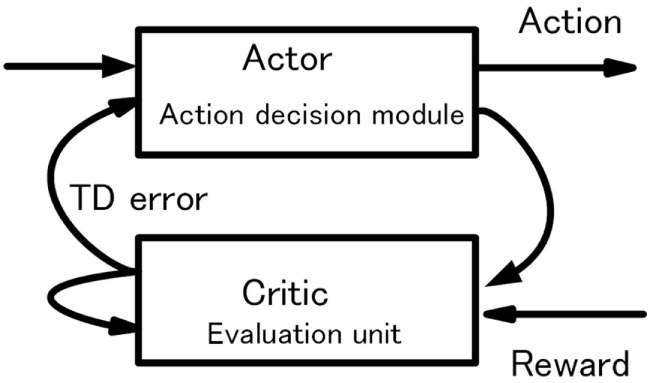
Actor–critic method in the basic-system.

The basic behavior of the robot consists of repeated processes of object search and reward acquisition. The robot walks randomly when there is no object in sight. When one or more objects are captured, the robot selects one preferred object and acts for it as mentioned previously. We call the object selected as desirable object–action pair in action decision phase hereinafter as “target.” The target corresponds to the object selected by the robot as an action target or objective. As shown in Figure 9, learning for action decision of the robot is performed as a reinforcement learning based on an actor–critic method. The action decision module selects an action as the actor, and the evaluation unit evaluates the action as the critic.

A chain of actions starting from selecting a target object to receiving a reward is taken as one learning episode to which reinforcement learning is performed. This chain of actions is hereinafter referred to as an “event.” The term “event” corresponds to the term “episode” commonly used in reinforcement learning, but to avoid confusion with the episodic memory, this article will use the term “event.” The robot can handle multiple objects simultaneously, but for simplicity the robot can select up to one target at a time.

When the object is selected as a target, the evaluation unit calculates the value $E^{t}\left({\text{A}}_{\text{O}{\text{b}}_{i}}^{*t},{\text{L}}_{\text{O}{\text{b}}_{i}}^{*t}\right)\;$ based on the attribute, position of the object by using a value function composed of a neural network. As both the targeted object and not targeted objects are affect the action decisions, even after an object is selected as the target, the robot is not necessarily bound by the targeted object until the reward is received. If more attractive or dangerous objects appear, the robot may change the target to deal with the new object. When the target is switched, the robot starts learning as a different event.

When the robot selects an action for the targeted object, reinforcement learning is performed based on the value $E^{t}\left({\text{A}}_{\text{O}{\text{b}}_{i}}^{*t},{\text{L}}_{\text{O}{\text{b}}_{i}}^{*t}\right)$ as follows:

(7) $$\Delta E\;\left(t\right)=E^{t-1}\;\left({\text{A}}_{\text{O}{\text{b}}_{i}}^{*t},{\text{L}}_{\text{O}{\text{b}}_{i}}^{*t}\right)\;+R_{\text{real}}\;\left(t\right)\;-\;E^{t-1}\;\left({\text{A}}_{\text{O}{\text{b}}_{i}}^{*t-1},{\text{L}}_{\text{O}{\text{b}}_{i}}^{*t-1}\right)\;,$$

(8) $$E^{t}\;\left({\text{A}}_{\text{O}{\text{b}}_{i}}^{*t-1},{\text{L}}_{\text{O}{\text{b}}_{i}}^{*t-1}\right)=\alpha \Delta E\;\left(t\right)+E^{t-1}\;\left({\text{A}}_{\text{O}{\text{b}}_{i}}^{*t-1},{\text{L}}_{\text{O}{\text{b}}_{i}}^{*t-1}\right).$$

Here, ${\text{A}}_{\text{O}{\text{b}}_{i}}^{*t}$ and ${\text{L}}_{\text{O}{\text{b}}_{i}}^{*t}$ indicate the attribute and the position of the selected object, and $E^{t}\left({\text{A}}_{\text{O}{\text{b}}_{i}}^{*t},{\text{L}}_{\text{O}{\text{b}}_{i}}^{*t}\right)$ indicates the evaluate value of the selected object at *t*. *R*_real_(*t*) indicates the real reward at *t*. Δ*E*(*t*) in Eq. 7 shows the prediction error in temporal difference learning at *t*. Based on this prediction error, the critic function performs learning as a neural network in the postprocessing phase using learning coefficient α, as shown in Eq. 8. If the prediction error Δ*E*(*t*) is positive, it corresponds to pleasant state or satisfaction with a reward above expectation, and in the negative case, unpleasant state or disappointment with less than expected reward. The above is a case where the robot does not change targets. However, if an object other than the target is selected, it is regarded that the event has been interrupted and the processes in Eqs 7 and 8 are not performed. When the target is switched, the robot starts learning as a different event.

### Learning in Cooperation with the Episodic Memory

Learning of the actor composed of a recurrent neural network is not as simple as the critic. The learning is executed in the following two stages using episodic memory, in awake-mode and in sleeping mode. (In the awake-mode, the robot is powered on and can react to external stimuli.)

#### Writing to the Episodic Memory in Awake-Mode

During awake-mode, the system writes a set of information (referred to as records) related to learning to the episodic memory during each postprocessing phase. The content of the record is composed of the position, attribute, action, output of the value function, and prediction error of the selected object. A sequential chain of records is recorded as a single “event” in the episodic memory. Later, reading the records is done sequentially.

#### Learning of Recurrent Neural Network in Sleeping Mode

In the sleeping mode, the system reads records from the episodic memory, and learning of the recurrent neural network as the actor is executed using the contents of the records as follows:

(i) The system preferentially selects an event including records with a relatively large prediction error and sequentially reads the records in the event.
(ii) The system changes the coefficient $b_{\mathrm{ij}}^{t}\left({\text{A}}_{\text{O}{\text{b}}_{i}},{\text{L}}_{\text{O}{\text{b}}_{i}},{\text{act}}_{j}\right)$ of the pattern detector for each record based on the following formula calculated by the information on the record
  (9) $$b_{\mathrm{ij}}^{t}\;\left(\;{\text{A}}_{\text{O}{\text{b}}_{i}}^{*t-1},{\text{L}}_{\text{O}{\text{b}}_{i}}^{*t-1},{\text{act}}_{j}^{*t-1}\;\right)\;=\;\beta \Delta E\left(\;t\right)+b_{\mathrm{ij}}^{t-1}\;\left(\;{\text{A}}_{\text{O}{\text{b}}_{i}}^{*t-1},{\text{L}}_{\text{O}{\text{b}}_{i}}^{*t-1},{\text{act}}_{j}^{*t-1}\;\right)\;,$$
  where β is a learning coefficient. Here, only the part of the recurrent neural network related to the above equation is activated, and the coefficient $b_{\mathrm{ij}}^{t}\left({\text{A}}_{\text{O}{\text{b}}_{i}},{\text{L}}_{\text{O}{\text{b}}_{i}},{\text{act}}_{j}\right)$ is changed in the direction along Δ*E*.

The reasons that the learning of the actor using episodic memory is performed during sleeping mode are as follows:

- To execute the learning shown in Eq. 9, it is necessary to activate only the part of the recurrent neural network related to learning. Other parts of network cannot operate at the same time. If the recurrent neural network learns during awake-mode, the network must temporarily stop responding to external stimulus during the learning process. The robot operation will have to stop intermittently during learning. Assuming the robot was an animal, it will not be able to react to dangerous conditions quickly if it tried learning while it was awake.
- Utilizing learning information after recording in episodic memory has some advantages. One is that the system can learn efficiently by utilizing experiences, based on selection, or repeating large impact events by looking back on past experiences. The other is the system enables relatively stable adaptation with less risk of over-training by not learning immediately when an event occurs.

In the case of an animal, execution of the learning in sleeping mode causes the animal to be in relative risk against predators during sleep, but overall there is merit for the animal to learn during sleep.

## Consciousness in the Basic-System

### Basic Hypothesis on Consciousness on the Basic-System

Consider the system-level-shared-information shown in the basic-system from the viewpoint of animals. We presume that an animal’s brain is composed of (a) functions that respond automatically or semi-automatically according to stimuli and (b) functions for system-level processing such as action decisions. The automatic or semi-automatic functions operate in parallel under loose coordination.

When an animal acts as one individual or one system, such as when going toward a prey or escaping from a predator, it is necessary for these functional units in the brain to have tightly related cooperation based on system-level decisions. For this purpose, it is an effective way to share consistent and clear information of objects and directions of action, such as approach or avoidance among functional units which should be tightly related for cooperation at the time. Based on this shared information, each functional unit performs consistent simultaneous operation so that the animal’s ability can be demonstrated as much as possible. In particular, “pleasant/unpleasant” is basic information that indicates either the necessity of action as individuals, approach or avoidance, and needs to be notified as quickly as possible. By using this pleasant/unpleasant information and object information in combination, to move more closely to prey or avoid predator becomes possible for the brain.

A unicellular paramecium backs away when it hits an obstacle ahead and swims at a speed that is more than twice the usual against a stimulus from behind. At that time, Paramecium sends information concurrently to thousands of cilia of Paramecium, organs of for move, by changing in membrane potential or ion concentration, in accordance to the stimulus received by the sensor. With this information, a large number of cilia perform a consistent operation along the direction of movement of the paramecium, as one individual (Kutomi and Hori, 2014). This indicates that even if the organism is extremely simple, if it is composed of many functional units and prompt action is required, to send system-level-shared-information to related organs all at once is necessary.

Although the contents of our “awareness” at each moment are diverse and contain various subtle elements, one of the main contents of awareness is the perceived world around us composed of objects, and the feeling of our “self” in this perceived world. Based on the above, we hypothesize that the main part of what we recognize as phenomenal consciousness corresponds to system-level-shared-information in the basic-system. We assume that even primitive animals have “awareness,” to adapt autonomously as a single system, and execute information transmission and processing corresponding to system-level-shared-information.

In addition, since an animal mainly acts using automatic or semi-automatic functions, system-level-shared-information is issued only when an action decision as a whole system is needed. If functions that are automatically or semi-automatically operated in parallel can respond appropriately to stimuli, system-level-shared-information is not issued. When we ride a bicycle for the first time, we are initially aware of the operations required to ride a bicycle, including pedaling, steering, and balancing. But when we get used to riding a bicycle, we are not aware anymore of the individual operations. Initially, the bicycle riding operations become the objective of the system-level action decision. As the semi-automatic processing function begins to work, the necessity to operate a bicycle disappears at the system-level. At this time, the system-level-shared-information for riding a bicycle is not required and is not generated anymore.

### Logical Organization of Consciousness and Self

**Figure 10:**
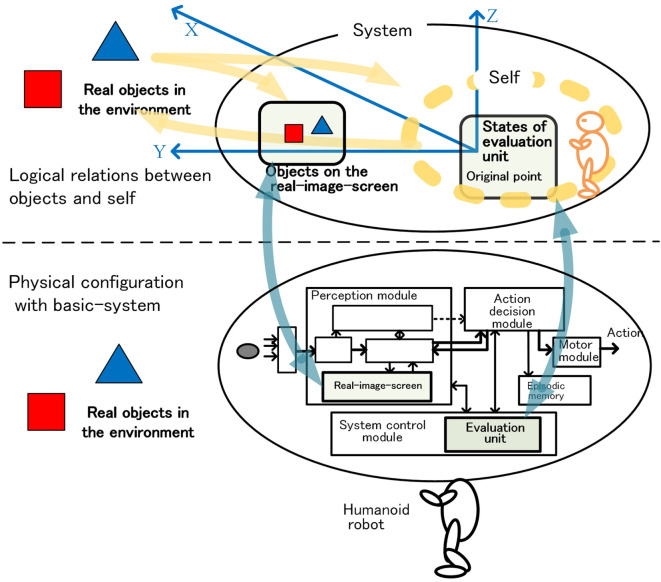
Logical relations between objects and self on the basis of the physical configuration.

Figure 10 shows the perceived space logically composed of the system-level-shared-information. In this space, the state of the evaluation unit and objects are the main elements. An evaluation unit located at the origin of the space evaluates the object. The relationship between the object and the robot including the system is the basis of the operation of the autonomous adaptation. Each object had been treated as a bundle of attributes etc. as we have mentioned. However, since the robot itself is composed of a large number of entities, the relationship between the robot and the object cannot be briefly expressed unless the robot is bundled too, or represented by something.

Since the robot operates on a complex interaction of various motor and functional units, bundling of some specific entities is not appropriate. Between the robot, and the object, “what kind of action the robot is going to do with respect to the object” is important, and “what is the bundled unit as the entity of the robot” is not necessarily required. From this point of view, the state of the evaluation unit briefly and basically shows the direction of action decision as a robot to the object, and implicitly represents the robot including the system itself.

Based on this view, we regard the state of the evaluation unit as a kind of “self” as in the bundle theory of self by Hume (Pike, 1967; Smith, 2017). The “self” existing at the origin forms the relationship between “self” and the objects. The “self” sees and copes with the objects. We speculate that this relationship contributes to the awareness of the first-person perspective as if the homunculus in our brain sees the outside world (the orange robot in Figure 10).

### The Binding Problem and the Delay Time of Libet’s Experiment

**Figure 11:**
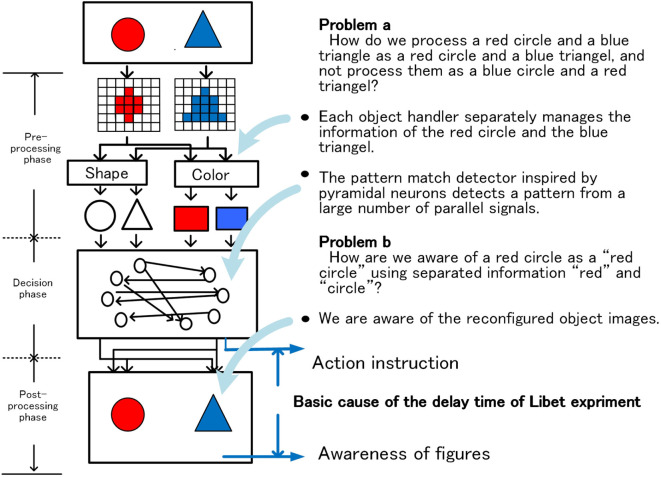
The binding problem and basic cause of the delay time of Libet’s experiment.

Based on the above hypothesis, the Binding problem and the delay time of Libet’s experiment can be accounted for as follows. An outline is shown in Figure 11.

#### Binding Problem

As Figure 11 shows, the brain is known to process shapes and colors with different functional units. In the case of a red circle and a blue triangle, shape and color are processed as separate signals by separate functional units. In relation to this, there is an unsolved problem, known as the Binding problem, in the brain (Kahneman and Treisman, 1992; Pylyshyn, 2001; Meyer and Damasio, 2009; Xu and Chun, 2009; Bays et al., 2011; Feldman, 2013). The binding problem is roughly expressed as the following two problems.

Problem a. How do we process a red circle and a blue triangle as a red circle and a blue triangle, and not process them as a blue circle and a red triangle?

Problem b. How are we aware of a red circle as a “red circle” using separated information “red” and “circle”?

Based on the hypothesis that the main contents of awareness correspond to system-level-shared-information, the system provides the answer to the problems as follows.

In the system, each circle and triangle is allocated to different object-handlers as different objects and managed. The object-handler instructs the related functional units to recognize (pattern recognition, color recognition, etc.) the allocated object and maintains the resultant signal as a set of parallel signals composed of shape and color. Information on the shape and color of the red circles keeps held by the object-handler until the object disappears. Information of each object is input to the action decision module as a set of parallel signals under the control of the allocated object-handler. In the action decision module, there are many action nodes corresponding to the type of various actions. And each action node has a lot of detectors that detect matched parallel signal pattern from the thousands of parallel signals. Using this function, each action node detects only the signal pattern that the corresponding action is deemed necessary from the thousands of parallel signals, and reacts to the signal pattern. This means that, in the case of animals that eat, for example, red apples but not blue prunes, the node for eat has a detector that detects “red” and “apple.” That is, although information of the shape and color of an object are processed separately, the object-handler manages it as a parallel signal belonging to the same object. Furthermore, a large number of parallel signals are directly checked for action decision while being parallelized using the mechanism inspired by pyramidal cells in the cerebrum. This makes it possible to explain the Problem a.

Furthermore, after deciding the action, the recognition system operates in the reverse direction to reconfigure the “red circle” on the real-image-screen, using the information, “red” and “circle” in the allocated object-handler. Then, we are aware of the “red circle” on the real-image-screen as a part of system-level-shared-information. In this way, the Problem b can be explained.

When combining and processing parallelized signals, the timing adjustment between signals is required. Without timing adjustment, it is not possible to perform processing based on the mutual relationship between signals appropriately. In general, when the number of stages of timing adjustment increases, the response time of the system becomes long because it is necessary to wait the signal arriving at the latest and to spend time to process signals at each processing stage. From this point of view and basic condition (ii), we speculate that the system uses a method in which the number of stages of timing adjustment relating to slow response is minimized.

#### The Time Delay in Libet’s Experiment

Famous experiment of Libet shows that our intentional movements are initiated before we become conscious to act, and have been calling a lot of debate so far (Libet, 2004). As we have repeatedly mentioned, in the system, since high priority is given to quick responses, action instructions are issued immediately after the decision phase and “awareness” occurs late in the postprocessing phase. The time difference shown in Figure 11 does not accurately correspond to the delay time indicated by the experiment of Libet, but we consider that it shows a basic cause of the delay time. From this viewpoint, we consider that our hypothesis for phenomenal consciousness is consistent with the Libet’s experiment.

## Proposal of the Extended-System

We have proposed the basic-system as an autonomous adaptive system that performs habitual behavior. In relation to consciousness, we have shown that awareness is an important operation for executing parallel processing. However, the basic-system cannot perform “goal-directed behavior,” consisting of setting a goal and conducting actions to achieve that goal through various attempts. Also functions that manipulate recollected objects, which are an important element of our conscious experience, are not incorporated. To realize these functions, we propose the “extended-system” as an extension of the basic-system.

### Outline of Goal-Directed Behavior in the Extended-System

#### Action Suspending

In the basic-system, an action instruction selected for the object is immediately executed in the decision phase, and the evaluation process for the action is executed in the postprocessing phase. In the extended-system, if necessary, the action on the object is suspended and no action is taken. For example, in cycle *t*, without taking action, the system predicts the reward of action for the object. Then, in cycle *t* + 1, the system can decide an action considering the predicted reward. This example shows that if the system temporarily suspends an action instruction, adaptive action considering multiple cycles becomes possible. We presume that this suspension of an action is related to “*the ability to delay immediate gratification for the sake of future consequences*” of children in the marshmallow test in psychology (Mischel, 2014).

#### Fast Decision and Slow Decision

In the extended-system, an action decision aiming for quick response (fast decision) and an action decision aiming at a higher level of adaptation with slow response speed (slow decision) are used depending on the situation. In the fast decision, the basic function corresponding to the basic-system operates with quick response, based on reinforcement learning. On the other hand, in the slow decision, the extended-system takes the risk of putting real actions on hold, allowing the system to aim for a higher level of reward.

When operating in slow decision, more processing time and resources in the system are used than in fast decision. We surmise that in the slow decision, the system needs some kind of “motivation” to take risks, use higher resources, and to try to achieve higher rewards. In the extended-system, in addition to the pleasant/unpleasant state of the basic-system, a value corresponding to motivation is maintained and managed as an indicator (degree of motivation), and execution of slow decision is controlled according to this value.

#### Assumed Primitive Behaviors

**Figure 12:**
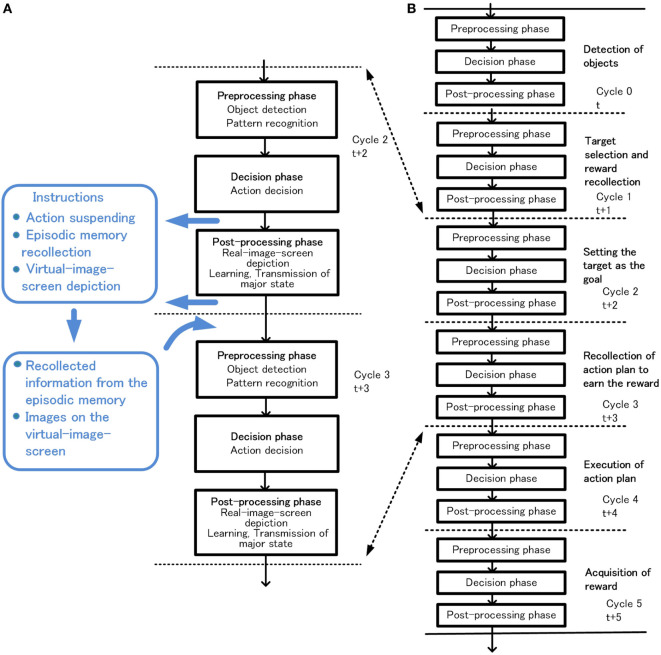
Outline of behavior in the extended-system. Coordination between cycle 2 and cycle 3 utilizing action suspending, episodic memory recollection or virtual-image-screen depiction **(A)**, and an example of goal-directed behavior **(B)**.

The goal-directed behavior consists of a chain of slow decisions aimed at achieving the goal. A primitive example of a series of decisions from detection of objects to acquisition of reward through various actions is shown in Figure 12.

### Configuration and Functions of the Extended-System

To configure the extended-system as simple as possible, the following policies were adopted:

- The extended-system is constructed using the functions of the basic-system as much as possible. Additional functions are minimized.
- The function to be configured as a new circuit is limited to functions commonly used, or functions requiring high speed.
- Information for high-level or detailed behavior is stored in long-term memory as much as possible and read out as necessary.

Based on these policies, when a goal-directed behavior is performed, although the number of times of reference to long-term memory and response time increases, it is possible to achieve a sophisticated adaptation at low cost. In addition, the time required for learning can be shortened as compared with the case of using a dedicated neural network circuit. This is because it is possible to record to long-term memory in a short time as compared with learning time of the dedicated neural network circuit.

**Figure 13:**
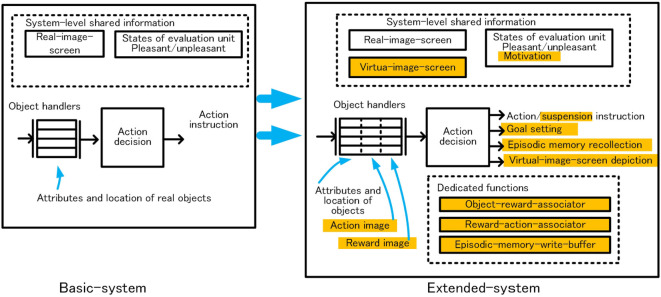
Outline of functional extensions in the extended-system.

**Figure 14:**
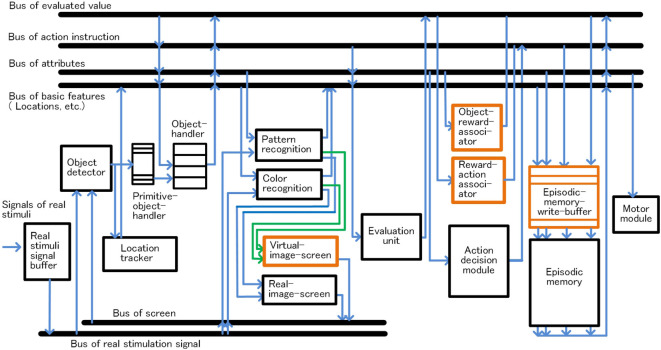
Bus configuration of the extended-system.

Under the above policies, the following dedicated functions were provided. Outline of functional extensions in the extended-system is shown in Figure 13. The bus configuration of the extended-system is shown in Figure 14. The orange box in the Figure 14 shows the main unit added to the basic-system.

#### Extension of Basic Functions in the Basic-System

The following functions are expanded in the extended-system.

##### Extension of Object Handling Function

In the extended-system, the goal is expressed by three elements, (1) what is targeted, (2) what actions to be taken on that object, and (3) what can be earned as reward. A single object-handler can hold this set of object, action, and evaluation value to express a goal. It is not necessary that all three elements of object, action, evaluation value is available.

##### Extension of Action Decision-Related Functions

The output of the action decision module was only an action instruction in the basic-system, but in the extended-system, instructions for suspending action, setting as a goal, recalling of long-term memory, and handling images as an object are added.

#### Addition of Image Manipulation Function

Functions related to the manipulating image, information generated inside the system, are added as common functions.

##### Buffer Memory for Expressing Patterns of Images (Virtual-Image-Screen)

To manipulate information generated within the system, such as recollected objects, in the same way as information of real objects existing actually at that time, a temporary buffer memory for expressing patterns of images, which we named “virtual-image-screen,” is provided. We call reconfigured information not corresponding to a real object existing in the environment at that time as a “virtual-image.” The “virtual-image-screen” is a kind of short-term memory, which maintains the virtual-images. Recollected contents from the long-term memory are depicted in the virtual-image-screen when in the awake-mode. Object-handlers can capture objects in the virtual-image-screen similarly to capturing objects in the real-image-screen, so objects captured from the virtual-image-screen can influence action decisions same as real objects in the basic-system. Based on this method, the extended-system can decide actions using past experiences or knowledge in the awake-mode.

The virtual-image-screen corresponds to our mental imagery as shown below. We use “mental imagery” as defined by Kosslyn (1994).

- In the postprocessing phase, objects on the virtual-image-screen are reconfigured using the attribute of objects same as with the objects on the real-image-screen. The contents of the virtual-image-screen are subject to object detection like the real-image-screen. In addition, the contents of the virtual-image-screen are transmitted to a wide range of the system as a component of system-level-shared-information.
- The signals for expressing the virtual-image-screen which are output from the recognition units (green lines in Figure 14), are not compared with the real stimulus by the autoencoder. In the case of the real-image-screen, comparison with real stimulus is executed by the autoencoder, so the system can express images on the real-image-screen clearly. In the case of the virtual-image-screen, the reconfigured images are blurred because there is no comparison with real stimulus. The extended-system uses a bus in which meaning is managed by the reference node in common with external stimuli. Thus, internally generated contents can have the same meaning corresponding to external stimuli.

By this configuration, the extended-system can treat the clear contents corresponding to real things in the real-image-screen, and the blurred contents generated inside the system in the virtual-image-screen concurrently. These contents correspond to how the human brain is aware, using a clear image of the real world and a blurred mental imagery.

##### Action Control by Action Patterns

Recollecting and execution of action in the extended-system is realized as a function to connect action instruction signals with visual action patterns. Visual action patterns are represented as a kind of image in the virtual-image-screen and manipulated as an object. When the system outputs an action pattern on the virtual-image-screen, the connected action signal is excited, and the action decision unit selects the corresponding action with highest priority. This means that the extended-system can be directed to perform that action by outputting a certain action pattern on the virtual-image-screen. In addition, the system can deliver the visual action pattern to the next cycle as part of the system-level-shared-information. In this manner, actions are treated as objects represented by a kind of visual action patterns. This function was adopted on the basis of findings of the mirror neuron (Rizzolatti et al., 2014).

##### The State of Evaluation Unit for Manipulating Reward

For the extended-system to handle reward as a goal, it is necessary to manipulate the state, such as pleasant or unpleasant, as a kind of object or signal independent of the system’s own evaluation unit state. The evaluation unit of the extended-system can have the following two states at the same time.

###### Effective-Excitation (EE) State

Reference nodes corresponding to the state are activated, and the activation is transmitted to the whole system. System-level learning is executed based on this state.

###### Non-Effective-Excitation (non-EE) State

Reference nodes corresponding to the state are activated, but the activation is not transmitted to the whole system and effective only as signals representing information of the state. The system-level learning is not executed based on the state. Signals of non-EE state are used for handling reward such as goals.

#### Object–Reward and Reward–Action Associating Function

Dedicated circuits, episodic memory write buffer, object–reward-associator, and reward–action-associator are provided to execute reward prediction from the target object and desirable action recollection from reward. The episodic memory write buffer maintains recent results of the action decision module for a 100 cycles before storing the episodic memory. The object–reward-associator, consisting of a bidirectional pair of neural networks, associates a target object and a reward value. Likewise, the reward–action-associator, consisting of a bidirectional pair of neural networks, associates a reward and an action.

The learning of object–reward-associator is performed by simultaneously exciting a target object and reward information on episodic memory write buffer in pairs, and supplying pairs of inputs and outputs to the unit through the bus. The neural network modifies the weight so that the supplied signal pair is associated with each other. In the reward–action-associator, learning is executed in the same way. These learning operations are executed under time shared control within the postprocessing phase.

### Extended-System Operation

Table 1 shows an example of robot operations with the extended-system for a goal-directed action, including the changes of state in system-level-shared-information corresponding to the robot awareness. This example was modeled with reference to the goal-directed behavior experiment using monkeys by Matsumoto et al. (2003) and the Experimental Cognitive Robot by Haikonen (2012).

**Table 1:** An example of robot operations as a minimal level goal-directed action.

Operations of the robot	Operations in the system	Awareness of the robot system-level-shared information			
		Real-image-screen (real-IS), virtual-image-screen (virtual-IS)		Evaluated value Effective excitation (EE), non-effective excitation (non-EE)	
		Real-IS	Virtual-IS	EE	Non-EE
**1. Searching objects**	a. Walking randomly for searching objects is installed as a basic function				
The robot detects nothing in the environment, then walks randomly to detect objects

**2. Detection of objects**	a. Two object-handlers maintain information of the 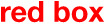 and 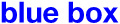 , respectively, and these are recognized	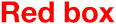 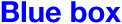			
The robot detects a 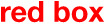 and a 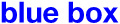 as object, then stops walking
	b. Reconfigured information of these boxes is depicted on the real-image screen			

**3. Target selection and reward recollection**	a. The action decision module selects the 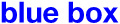 as a target, then object–reward-associator outputs reward recollection related to the 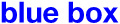	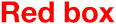 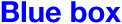			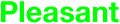
The robot selects the 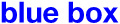 as a target and recollects reward related to the target
	b. State of the evaluation unit becomes 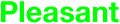 in **non-EE**				
	c. No actual action instruction			

**4. Setting the target with reward as the goal**	a. The object-handler allocated to the 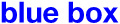 maintains information of reward as a goal	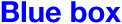			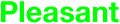
The robot sets the 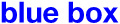 including reward as the goal
	b. No actual action instruction			

**5. Recollection of action to earn the reward**	a. The behavior of the action, output of the reward–action-associator, are depicted in the virtual-image-screen	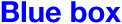	**Touching action**		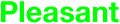
	b. No actual action instruction			

**6. Execution of action plan**	a. The robot is charged by touching the 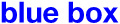	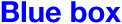		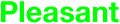	
The robot touches the 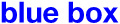
	b. The evaluation unit becomes 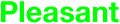 in **EE**				

**7. Acquisition of reward**
The robot is 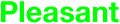 by charge really		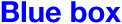		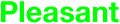	

**8. Execution of learning through these experiences**	a. Learning is executed in the sleeping mode	–	–	–	–

*Significant states and objects are depicted in color or bold (for highlighting purposes)*.

The robot determines actions based on the conscious information in the previous cycle and summarizes the result to the next conscious information. This process is repeated. Defining that the perceived world for the robot is the world of what the robot is aware or conscious of, from the viewpoint of the robot, the robot decides and acts on the world using summarized conscious information. We think that this flow of state changes in the system-level-information corresponds to the flow of consciousness for us humans.

In Table 1, it was assumed that the motivation level of the robot is sufficiently high for performing the slow decision. If the robot is exhausted and the motivation is lowered, the robot ignores the detected objects. When the robot receives rewards, the system evaluates the reward as pleasant/unpleasant according to the difference between the expected value and the obtained value. Primitive learning is performed by reflecting this value (pleasant/unpleasant) in the learning of the object–reward-associator and the reward–action-associator. However, learning methods throughout the entire robot including motivation adjustment are still under consideration.

## On Consciousness

### Consciousness as Awareness

We assumed that the brain basically works like the basic-system in principle because the brain should perform at its full potential as a parallel-processing system. In this case, the brain selects and decides the fastest and most efficient action, and responds immediately. After the action decision, postprocessing is done throughout the brain and prepared for the next stimulus.

In this postprocessing, scattered information is organized/integrated, learning based on reward is executed, and these results are notified through the brain. The phenomenon of awareness corresponds to the most important notified information that is “system-level-shared-information,” composed of states of evaluation unit and objects. This information forms a space where the state of evaluation unit is located at the origin and various objects exist together. This space corresponds to the “subjective space” that we are aware of on a daily basis, and the evaluation state corresponds to “self.”

One of the characteristics of phenomenal consciousness is “integration of information.” Tononi (2012) explained in the integrated information theory using Φ, but we consider that Φ is unnecessary for explanation of consciousness. Through the processing of the decision phase and postprocessing phase shown so far, “integration of information” as a phenomenon can be generated. Based on our model, we can explain the binding problem and show the basic causes of delay in Libet’s experiment, which indicates that Φ is unnecessary. Consciousness is a necessary function for the brain to perform at the full potential as a central control system of an animal.

Our proposed system is close to Haikonen’s robot and Franklin’s system (Haikonen, 2012; Franklin et al., 2014), and to proceed in the future, it is necessary to incorporate the various functions proposed in these systems. However, our proposed system is different from their methods in the core design regarding action decision and consciousness. In GWT, dedicated processors compete for the right in the limited storage area called Global Work Space, and the action plan of the processor that got this right is broadcasted and conscious. In HCA, dedicated processors attempt to communicate with each other, and the main successful communication becomes conscious. For the following reasons, our proposal is more appropriate than GWT and HCA.

(i) In GWT and HCA, the information and actions to be selected are determined by mutual relationship among individual dedicated processors. We think that reflections of what is desirable as a system are not sufficient in this selection. In our model, the system chooses the optimum pair based on the desirability as a system from “object and action pair.” The system further performs reinforcement learning using episodic memory for individual combinations. In addition, in our model, it is a choice of optimal object and action pair, so multiple pairs cannot be allowed to exist simultaneously. This explicitly explains the “unity” which is the basic characteristic of consciousness (Brook and Raymont, 2017).
(ii) In GWT, it is claimed that broadcasting is the main factor of awareness or consciousness. We presume this broadcast correspond to wide-area transmission in postprocessing in our model. However, in postprocessing, various information that we are not phenomenally conscious of is simultaneously transmitted in a wide area. We assume that we are only aware of system-level-shared-information, not simply the information transmitted over a wide area. The system-level-shared-information is composed of the state of the evaluation unit and the state of the object. We speculate that activation of the evaluation unit that represents “self” is indispensable factor of conscious experience.
(iii) Dehaene and Changeux (2011) assert the validity of GWT based on brain observations such as fMRI, event-related potentials. However, since our model shown in this article is expected to be observed as a phenomenon similar to GWT, it also supports the validity of our model. In our model, the recurrent neural network optimization process in the decision phase roughly corresponds to the activation centered on the frontal lobe, and the processing in the postprocessing phase roughly corresponds to the activation of a wider area including the occipital lobe.

### Consciousness as an Important Function for the Complex Brain

In the extended-system, we think that the chain of “conscious information,” which directs actions toward a goal with intention, corresponds to our “proactive” conscious state. In addition, it is important that the conscious information can be handled as objects in the next cycle. Since the conscious information expresses the state of the system in a summarized form, the system can decide an action efficiently and easily by using this summarized information. This shows that by manipulating the conscious information, complex systems such as the extended-system can be controlled efficiently and easily. We speculate that it is through this function of consciousness that we can “think” and make decisions without being aware of the complexity of the human brain.

## Discussion

### Duality Model

Duality models of human behavior, such as fast/slow thinking in the behavioral economics field and impulsive/reflective system in the social psychology field are well known (Deutsch and Strack, 2004; Kahneman, 2011). We predict that this duality arises from fast decision due to direct fast responses, and slow decision due to sophisticated adaptation at the expense of response speed, depending on the circumstances in the extended-system. An action mainly composed of fast decisions appears as a fast or impulsive action, and an action mainly composed of slow decisions appears as a slow or reflective action.

From another point of view, a fast decision shows passive and reactive behavior against the stimulus, such as prompt decision as to whether or not to eat when bait appears. On the other hand, in a slow decision, such as when a stimulus that is not directly related to bait has appeared, shows a proactive behavior that looks ahead toward an intended goal.

### Goal-Directed Behavior Incorporating a General-Purpose Computer Like Function

Although there are various ways to perform goal-directed behavior, the main aim of the proposed extended-system was “to realize advanced adaptation at a relative low cost by sacrificing response speed.” We assumed that it is important to realize goal-directed behavior through a combination of common or general-purpose circuits together with long-term memory as designed in the early computer EDVAC (von Neumann, 1945). We predict that a conscious autonomously adaptive system that achieve goals set by itself will become a powerful control system for the humanoid robots by incorporating a kind of von Neumann type computer as extended functions.

## Conclusion

We proposed a basic architecture of an autonomous adaptive system with conscious-like function for a humanoid robot. We think resembling the human brain at the level of the basic logical structure, architecture, is a meaningful way of designing a control system for a truly useful humanoid robot. Interaction or communication between humans and humanoid robots will be much easier if both sides shared the same behavior characteristics based on the same architecture, such as consciousness or duality. However, the proposal in this article currently remains at the architecture design level, and verification through simulation is still only partial. We plan to further refine the system configuration with reference to the results of previous research by Haikonen and Franklin et al., as well as new findings. Evaluation of the dynamic characteristics of the system through simulation is also planned.

## Author Contributions

YK designed the basic architecture of this autonomous adaptive neural network system. YK and KM together discussed and built upon the basic design to produce the complete architecture of the proposed system and together wrote this article.

## Conflict of Interest Statement

The authors declare that the research was conducted in the absence of any commercial or financial relationships that could be construed as a potential conflict of interest.
